# Evaluation of treatment outcomes in a 3 years post-graduate orthodontic program using the peer assessment rating (par)

**DOI:** 10.4317/jced.51512

**Published:** 2014-10-01

**Authors:** Paloma González–Gil-de-Bernabé, Carlos Bellot-Arcís, José M. Montiel-Company, José L. Gandía-Franco

**Affiliations:** 1 Assistant professor. Stomatology Department, Faculty of Medicine and Dentistry, University of Valencia, Spain; 2 PhD, Adjunct Professor. Stomatology Department, Faculty of Medicine and Dentistry, University of Valencia, Spain; 3 PhD. Post-Doctoral Assistant Professor, Stomatology Department, Faculty of Medicine and Dentistry, University of Valencia, Spain; 4 PhD. Tenured Lecturer, Stomatology Department, Faculty of Medicine and Dentistry, University of Valencia. Spain

## Abstract

Objectives: To maintain high treatment quality it is important to evaluate orthodontic treatment results using objective methods. Outcome assessments allow private practitioners and university students to evaluate their results and raise the level of treatment outcomes. The aim of this study was to assess the orthodontic treatment outcome in a post-graduate orthodontics program in the University of Valencia (Spain) and to determine whether the treatment outcome is related to several factors as gender, age at start of the treatment, treatment duration, treatment method, extraction-non extraction treatment and cooperation needed. Material and Methods: A sample of 50 patients treated in the post-graduate clinic was randomly selected. Pre-treatment and post-treatment study casts have been assessed by the Peer Assessment Rating (PAR index). The influence of various factors: gender, age at start of the treatment, treatment duration, treatment method, extraction-non extraction treatment, cooperation needed and number of students finishing each case, were statistically analyzed. Results: According to the PAR index, orthodontic treatment reduced the malocclusion in a mean point reduction of 21.4 (CI 95% 18.7-24.1) and a mean percentage reduction of 80.5% (CI 95% 75.9-85.1). The total of the cases improved, 44% of the patients were in greatly improved category. Conclusions: None of the variables studied influenced significantly the treatment outcomes regarding the PAR. Based on the general classification criteria of the index, the results showed that the patients received a high standard treatment.

** Key words:**Treatment outcome, orthodontics education, PAR Index.

## Introduction

To maintain high treatment quality it is important to evaluate orthodontic treatment results continuously, using objectives methods (1). Outcome assessments allow private practitioners and university students to evaluate their results and raise the level of treatment outcomes (1,2). Special importance is to keep the quality of educational in postgraduate clinics high, in order that students are exposed to optimal treatment procedures (3-8). By evaluating the quality of treatments, we evaluate the skills acquired by the students and, of course, the quality of a 3 years postgraduate orthodontic program.

Several indices have been developed to assess the success of treatment, to ensure uniform interpretation and application of criteria (9). The most commonly used to assess orthodontic outcome, is the Peer Assessment Rating Index [PAR]. It is an occlusal index designed to measure how much a patient deviates from normal align-ment and occlusion (10).

The PAR components are the upper and lower anterior segments, the upper and lower right and left segments, the overjet, the overbite and the midline. They generate summarize data about the malocclusion and return a numeric value between 0, corresponding ideal occlusion and 60 near the worst (11). The difference between final PAR and initial PAR reflects the degree of improvement and therefore the success of the treatment (8,12).

The PAR index has been used widely for evaluating the effects of treatment in a variety of circumstances: treatment with fixed and removable appliances (3), to compare treatment in orthodontics schools and private practices (3,4,7,8), results from different type of initial malocclusion (13) also in the first phase treatments (14) and in the assessment of orthognatic surgery outcome (15).

This index has been shown to have good intra and inter-examiner reliability and offers standardization in assessing the outcome of orthodontic treatment in three categories: Worse-no different [there is not reduction in the initial PAR score or less than a 30%], improved [greater than or equal to 30% reduction in the PAR score], greatly improved [the total PAR score is reduced more than 22 points or greater or equal to 70%] (12).

To allocated the outcome in a high standard treatment from the total patients, according to the general classification criteria of the index, the sample should have a higher percentage than 70% cases showing improvement, more than 40% should be of great improvement and cases with negligible minimum improvement or worse should be a maximum of 5%.

The objectives of this retrospective study were:

• To evaluate the quality of treatment performed in a postgraduate orthodontic program in the University of Valencia [Spain] using the PAR index.

• To determine whether the treatment outcome [change in PAR] is related to the following factors: gender, age at start of treatment, duration of treatment, treatment technique, extractions, cooperation needed and number of students treating a case.

## Material and Methods

The sample for this study was obtained from the patients treated with fixed appliances during the last ten years at the Master of Orthodontics at the Dental School of the University of Valencia. Fifty-five were randomly selected from a total of 440 cases in retention phase, five of them were excluded because they lacked completed records. The study obtained the approval, from the Institutional Review Board from the Stomatology Department [University of Valencia, Spain].

The following information was noted in each case from the records: gender, date of birth, date treatment started, date treatment finished, age at the start of treatment, duration of treatment, treatment technique [Standard, Bidimensional, MBT, Tip-edge, Smart-clip], extracted teeth, cooperation needed from the patient, and number of students treating each case. The PAR score was obtained from pretreatment and post-treatment study models and its components were: upper labial segment alignment, lower labial segment alignment, buccal segment relationship, overbite, overjet and midline.

The error of the method for recording of the PAR index was evaluated from double measurements, one month interval by the same examiner. Intraexaminer agreement was analyzed by the Intraclass Correlation Coefficient [ICC] in order to evaluate the validity of the method.

A further measurement of 20% of the cases, randomly selected, was evaluated for another examiner, an orthodontist calibrated at the Occlusal Index Calibration Course held by Dr. Richmond, author of the PAR index (11). Interexaminer reliability was also evaluated with the Intraclass Correlation Coefficient [ICC].

The outcome was assessed using the numerical and percentage reduction weighted PAR score.

To estimate the relationship between the variables and the PAR reduction and percentage change rates, nonparametric tests were made. The Mann Withney’s U test procedure was applied in the categorical variables with a *p*<0.05 significance level.

Pearson’s Correlation Coefficient was computed to assess the linear relationships between score reduction [total points and percentage] and the numeric variables evaluated [age at start treatment and time of treatment].

 All statistical analysis was carried out using the Statistical Package for Social Sciences for Windows software, version 21 [SPSS v. 21].

## Results

Out of the total sample of 50 patients, 38% were men [n=19] and 62% were women [n=31]. The average pretreatment age was 17.2 [CI 95% 14.4-20.0]. The average treatment duration was 2.4 years [CI 95% 2.1-2.7].

The treatment technique was 42% Standard, 18% Bidimensional, 14% MBT, 18% Tip-Edge and 8% Smart-clip. Extraction treatment was performed on 20% of patients. The number of students taking part in each case was: 56% one student per patient, 44% two or three. The need for cooperation by the patient was required in 78% of cases.

The pretreatment PAR index mean was 26.3 [CI 23.6-29.1] and the post-treatment PAR index mean was 4.9 [CI 3.6-6.3]. The mean value of the changes PAR was 21.4 [CI 18.7-24.1] and the percentages reduction was 80.5% [CI 75.9-85.1].

None of the categorical variables studied [gender, technique, extractions, number of master students and need for patient’s collaboration] influenced significantly the treatment outcomes regarding the PAR ( Table 1).

**Table 1:**
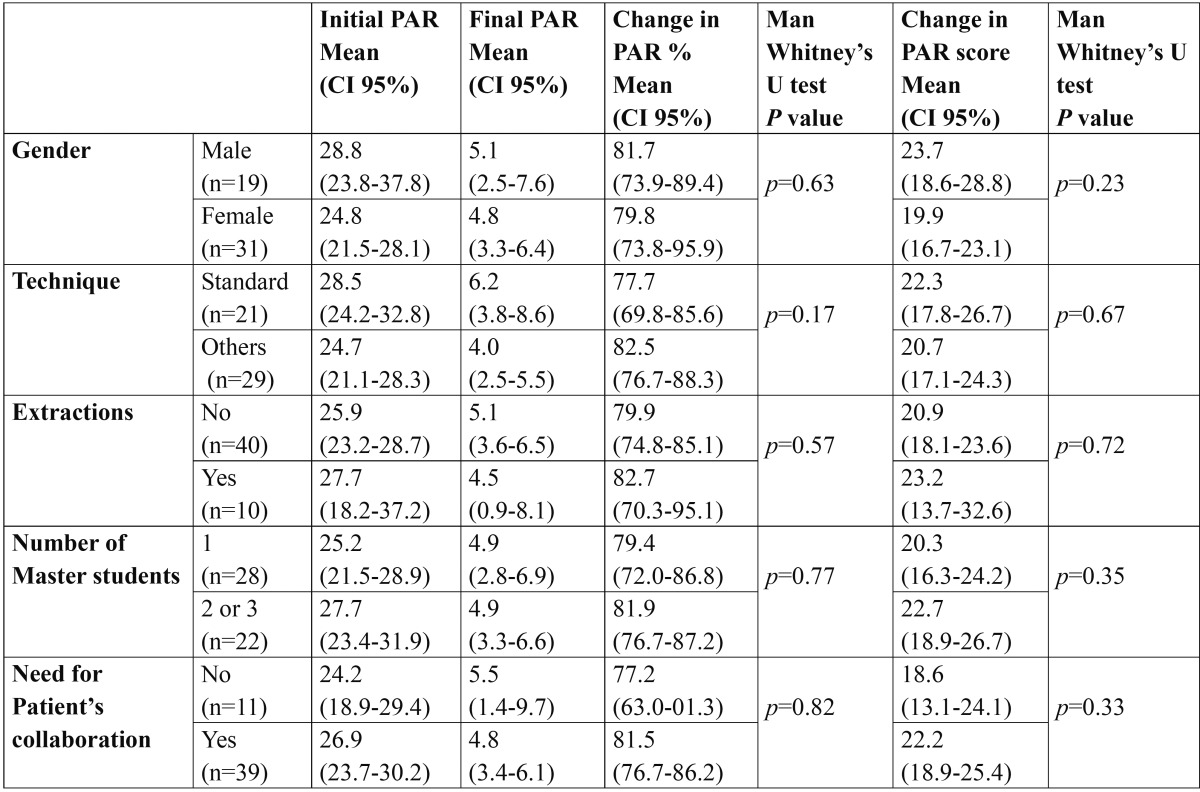
Mean PAR distribution related with categorical variables: gender, technique, extractions, number of Master students and need for patient’s collaboration. Statistical nonparametric test applied. *Significant differences (*p*<0.05) in Man Whitney’s test. CI-95% Confindence Interval.

In the quantitative variables we found statistical negative correlation between average treatment duration and change in PAR percentage, but this correlation was weak [Pearson =-0.28]. Treatment duration had also weak and negative correlation with age at the beginning of treatment [Pearson=-0.30].

**Figure 1:**
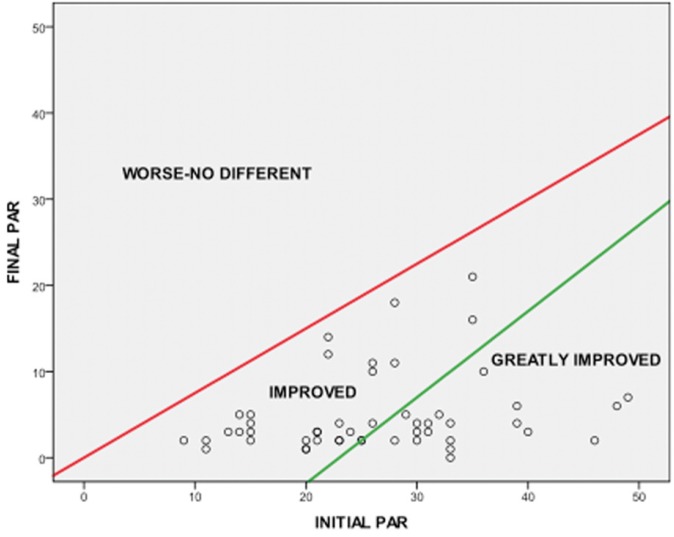
Pre-treatment and post-treatment PAR scores distribution in different categories.

To assess the success in our sample, figure 1 shows the degree of improvement classified into the following categories: 1. No improvement, 0 % of the cases; 2. Improvement , 56% of the cases , 3. Greatly improved, 44 % of the cases.

The Intraclass Correlation Coefficient showed excellent intra-examiner reliability: 0.98 for the initial and final PAR measurements. Excellent Inter-examiner agreement validity resulted [0.87] for the initial PAR and 0.89 for the final PAR, compared with de Gold Standard.

## Discussion

Our sample, in relation to other authors who have conducted the study in orthodontic schools, is quite similar in the percentage of men and women distribution (8,16). The results of the change and percentage PAR obtained does not vary with the sex of the patient in accordance with other researchers.

Although the average age at the start of the treatment in our sample was 17 years, higher than in other studies (2,5,16), 60% of our cases treatment was initiated between ten and fifteen years old. We found no association between starting age and treatment outcome.

The average treatment duration was 28 months and it agrees with other authors who carried out similar studies (4,6,17). We found statistical negative correlation between average treatment duration and change in PAR percentage, but this correlation was weak [Pearson = -0.28]. Some authors have found that the longer treatment produces worse outcomes (2,6), and others get totally opposite results (18). We cannot forget the risk of long-term treatment in terms of iatrogenic and we must distinguish cases that drag on for lack of cooperation from those who do it for a better finish.

Analyzed in our study the relationship of the results obtained and the different treatment techniques used, we found no statistical significance like others authors (19,20) but we could not compare with them because they use other indices to assess the outcome of treatment and only one of them analyzed two matching techniques (20).

The number of students taking part in the treatment did not influence the result in terms of quality of treatment outcomes as the study conducted in three orthodontic clinics by Cook *et al.* (7). Noted that the duration of Master study last for three years, so that, students can finish the largest number of cases and improve their training.

Another variable considered was the presence of extractions in treatment. Our percentage of extractions was 20% of cases, less than in other samples of random selection (6,16). We found no statistical relationship like other results consulted (21,22).

The need for patient’s collaboration was also analyzed in our study. It was necessary in 80% of cases without presenting statistical relationship with the results. Only one author associated this variable with treatment duration, but not with the treatment outcome (6).

In making our measurements, the findings show a high degree of predictability in agreement with other authors (2,9). The validation of our measures using a Gold Standard shows a very high concordance, somewhat higher than those found by other authors (22-25), perhaps due to both the author and the Gold Standard, have been studied at the same research clinic which may lead to unified observation criteria.

The quantitative variable for the initial PAR index was an average of 26.3 PAR similar to other authors (2,3,8,17). The quantitative average final PAR obtained in our study was 4.9, also similar to other studies (2,4).

The change in value of the PAR and the percentage change PAR were respectively 21.4 and 80.5%, obtained from the values initial and final PAR, allow us to compare with clinical training in orthodontics and see outcome studies percentage reduction between 81% and 70.7% on similar samples (2,24).

The limitations of the study were the size of the sample [n=50], but we should consider that it is enough to achieve the proposed objectives. Moreover, we collected a random sample representative of the whole cases treated at the post-graduate orthodontic program. Regarding the validity of the method, high values were obtained in both, the intraexaminer and interexaminer agreement.

The PAR index allows us to classify the overall level of treatment and rated data of the PAR value change and percentage change in varying degrees of improvement. That gives a set value to the results. In our sample, to sort the data, the results are clear and conclusive: all the cases improved and the 44% with great improvement. Others authors showed a 4% worsening and 55% great improvement (7) and 3% worsening and 50% big improvement (17).

According to the general classification criteria of the index, to consider a high standard of treatment, the sample should have a higher percentage than 70% cases showing improvement and more than 40% should be of great improvement and cases with negligible minimum improvement or worse should be a maximum of 5%. Our results clearly demonstrate meeting the criteria required of the high standard rating: 100% improved and from this total, 44% with great improvement.

By evaluating the quality of treatments performed by students using the PAR index, we evaluate the skills acquired by the students and, of course, the quality of a 3 years post-graduate orthodontic program.
